# Jumonji domain-containing protein 1A promotes cell growth and progression via transactivation of c-Myc expression and predicts a poor prognosis in cervical cancer

**DOI:** 10.18632/oncotarget.13208

**Published:** 2016-11-08

**Authors:** Jue Liu, Ming Zhu, Xue Xia, Yuliang Huang, Qunfeng Zhang, Xiaoxu Wang

**Affiliations:** ^1^ Department of Obstetrics and Gynecology, The Second Affiliated Hospital of University of South China, Hengyang, Hunan Province, 421001, P.R. China; ^2^ Department of Orthopaedics, The Second Affiliated Hospital of University of South China, Hengyang, Hunan Province, 421001, P.R. China; ^3^ Department of Obstetrics and Gynecology, Shanghai General Hospital, Shanghai Jiao Tong University School of Medicine, Shanghai 201620, P.R. China

**Keywords:** cervical cancer, JMJD1A, c-Myc, prognosis, immunohistochemistry

## Abstract

Jumonji domain-containing protein 1A (JMJD1A) plays a key role in the development and progression of several cancers. Here, we showed that the expression of JMJD1A is increased in cervical cancer cells and tissues, and that suppression of JMJD1A inhibits proliferation, migration, and invasion of cervical cancer cells. JMJD1A induced transcription of c-Myc, which is essential for cervical cancer growth and progression. Clinical data showed that JMJD1A expression correlated with lymph node metastasis (*P*=0.031) and FIGO stage (*P*=0.007). Increased c-Myc levels were associated with tumor differentiation (*P*=0.007) and FIGO stage (*P*<0.001). JMJD1A protein levels correlated with c-Myc expression (*P*<0.001), and high co-expression of the two proteins correlated with a poor prognosis. Survival analysis showed that JMJD1A and c-Myc levels are independent prognostic factors for cervical cancer patients. These results suggest that JMJD1A is a promising therapeutic target in cervical cancer.

## INTRODUCTION

Cervical cancer continues to be a leading cause of death among women in less developed countries including China [1, 2]. Many recent studies have focused on cervical cancer, leading to reduced mortality rates; however, the underlying molecular mechanisms remain unclear [3-6]. The diversity of pathological characteristics complicates the study of cervical cancer molecular mechanisms. Precision medicine may help identify effective treatments on an individual basis in the future [7-9].

Jumonji domain containing 1A (JMJD1A), also known as JHDM2A and KDM3A, is a histone demethylase that removes mono- and di-methyl groups from histone H3K9 (specifically, from H3K9me1 or H3K9me2) [10], thereby mediating transcriptional activation [11, 12]. JMJD1A acts on various target genes to regulate such processes as metabolism, spermatogenesis, stem cell self-renewal, sex determination, and differentiation [13-15]. Recent studies have also indicated that increased JMJD1A expression is associated with the development of different types of cancer, including renal cell carcinoma [16], prostate cancer [17], and liver cancer [18]. In addition, elevated JMJD1A levels have been associated with poor cancer prognosis [18-20]. However, the expression patterns and the potential prognostic role of JMJD1A in the pathogenesis of cervical cancer remain unclear. In the present study, we investigated the expression and function of JMJD1A in cervical cancer cells and assessed its prognostic significance in cervical cancer.

## RESULTS

### JMJD1A expression is increased in cervical cancer

**Figure 1:**
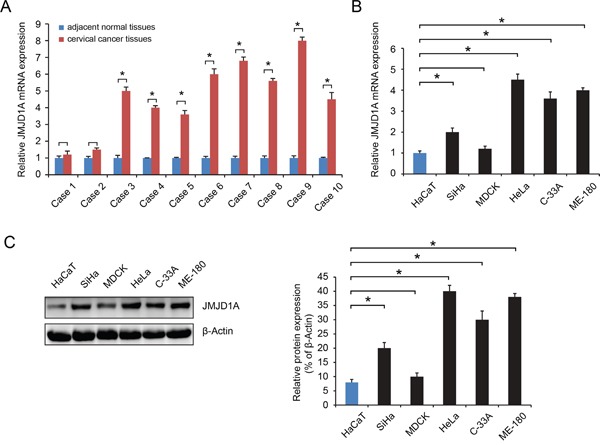
JMJD1A expression level is significantly upregulated in cervical cancer **A.** RT-qPCR was conducted to examine the expression levels of JMJD1A in 10 cases of cervical cancer tissues and their matched adjacent non-tumor tissues. JMJD1A expression was significantly higher in eight cases of tumor tissues than in matched paracancerous controls. **B** and **C.** RT-qPCR (B) and western blot (C) was employed to examine the expression levels of JMJD1A in five common human cervical cell lines, HeLa, SiHa, ME-180 and C-33A, as well as in a non-tumorigenic human epithelial cell line HaCaT. JMJD1A mRNA and protein expression was higher in cervical cell lines. We have set the mRNA and protein control levels to 1.0, relative to internal standards GAPDH. *P < 0.05.

We initially analyzed JMJD1A expression in samples of tumor and adjacent normal tissues from ten patients with cervical cancer. The results showed that in eight of the tumor samples, JMJD1A expression was increased as compared to matched paracancerous controls (*P*<0.05, Figure 1A). JMJD1A mRNA and protein levels in the cervical cancer HeLa, SiHa, ME-180, and C-33A cells were higher than in the non-tumorigenic human epithelial cell line HaCaT (*P*<0.05, Figure 1B, 1C). These data indicate that the JMJD1A expression is increased in cervical cancer.

### Downregulation of JMJD1A inhibits cervical cancer cell proliferation, migration and invasion *in vitro*

**Figure 2:**
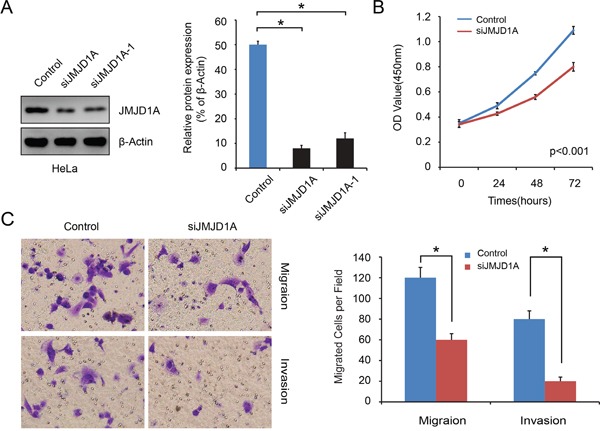
Stable downregulation of JMJD1A expression inhibits cervical cancer cell proliferation, migration and invasion *in vitro* **A.** Western blot analysis was performed to detect the knockdown effect on JMJD1A expression in HeLa cell. It was found that siJMJD1A achieved a high efficacy in silencing JMJD1A expression compared to the negative control. **B.** Effect of JMJD1A knockdown on proliferation was evaluated by Cell Counting Kit-8 assays. Suppressing JMJD1A significantly reduced cell proliferation in comparison with control. **C.** Transwell and Boyden chamber assay was conducted to examine cell migration and invasion ability. Knockdown of JMJD1A clearly inhibits cell migration and invasion of pancreatic cancer *in vitro*.*P < 0.05.

We used a lentiviral shRNA vector to stably knock down the expression of JMJD1A in HeLa cells, which have relatively high levels of JMJD1A (*P*<0.05, Figure 1B, 1C). Compared to negative control, silencing with siJMJD1A suppressed more than 80% of the JMJD1A protein expression (*P*<0.05, Figure 2A). Thus, stably transfected cells HeLa-conJMJD1A and HeLa-siJMJD1A were used in the following experiments.

We used CCK-8 assays to examine the effect JMJD1A expression on cervical cancer cell growth. The growth curves showed that suppression of JMJD1A significantly reduced cell proliferation in comparison with control (*P*<0.05, Figure 2B). Furthermore, Transwell and Boyden chamber assays showed that there were significantly fewer migrated cells in the siJMJD1A group than in the control group (*P*<0.05, Figure 2C). To eliminate the possibility that the decrease in cell migration and invasion in JMJD1A-suppressed cells was a secondary consequence of reduced cell proliferation, we placed serum-free medium in the upper chambers. These *in vitro* results indicate that JMJD1A knockdown inhibits cervical cancer cell proliferation, migration and invasion.

### JMJD1A regulates c-Myc transcriptional activity

**Figure 3:**
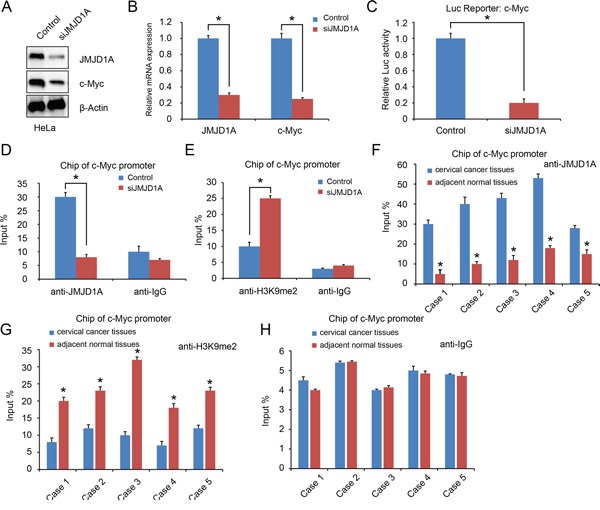
JMJD1A regulates c-Myc transcriptional activity **A** and **B.** JMJD1A knockdown dramatically decreased c-Myc expression at protein (A) and mRNA (B) level. **C.** HeLa cells were transfected with the indicated c-Myc promoter reporter containing JMJD1A-binding sites. The c-Myc promoter activity was normalized via co-transfection of a β-actin/Renilla luciferase reporter containing a full-length Renilla luciferase gene. The luciferase activity in the HeLa cells was quantified using a dual luciferase assay system (Promega) 24 hours after transfection. Renilla expression plasmid was used as an internal control. Knockdown of JMJD1A significantly inhibited the c-Myc promoter activity. **D** and **E.** Chip assay using chromatins prepared from HeLa-conJMJD1A and HeLa-siJMJD1A. Chip analysis was conducted on c-Myc promoter regions using the indicated antibodies. Enrichment was determined relative to input controls. (D). Compare to the HeLa-conJMJD1A group, reduced JMJD1A recruitment to the c-Myc promoter was found in HeLa-siJMJD1A. (E). Knockdown of JMJD1A increased H3K9me2 levels at the c-Myc promoter significantly. **F, G** and **H.** Chip assay using chromatins prepared from cervical cancer tissues and adjacent normal tissues. (F). Compare to the adjacent normal tissues, increased JMJD1A recruitment to the c-Myc promoter was found in cervical cancer tissues. (G). Enhanced H3K9me2 levels at the c-Myc promoter in cervical cancer tissues significantly. (H). Normal human IgG was used as a negative control. *P < 0.05.

An earlier study showed that JMJD1A stimulates prostate cancer cell proliferation by increasing c-Myc expression [21]. Our results showed that JMJD1A suppression decreased c-Myc expression in cervical cancer cells (*P*<0.05, Figure 3A, 3B). Using luciferase assays with transfected cancer cells (HeLa-conJMJD1A or HeLa-siJMJD1A), we showed that JMJD1A knockdown significantly inhibited c-Myc expression (*P*<0.05, Figure 3C). To further provide evidence that JMJD1A binds directly to the c-Myc promoter, we conducted a Chip assay in HeLa-conJMJD1A and HeLa-siJMJD1A cells. Compared to HeLa-conJMJD1A cells, HeLa-siJMJD1A cells exhibited a reduced JMJD1A recruitment to c-Myc promoter (*P*<0.05, Figure 3D). In contrast, knockdown of JMJD1A increased H3K9me2 levels at the c-Myc promoter (*P*<0.05, Figure 3E).

Next, we analyzed JMJD1A recruitment to c-Myc promoter in five paired cervical cancer tissues and adjacent normal tissues. Compared with normal tissues, in cervical cancer tissues, JMJD1A recruitment to c-Myc promoter was increased, while H3K9me2 recruitment was decreased (*P*<0.05, Figure 3F-3H). Taken together, these data suggest that JMJD1A binds to c-Myc promoter to mediate c-Myc transcription in cervical cancer cells.

### JMJD1A-induced cell proliferation, migration, and invasion is dependent on c-Myc in cervical cancer cells

**Figure 4:**
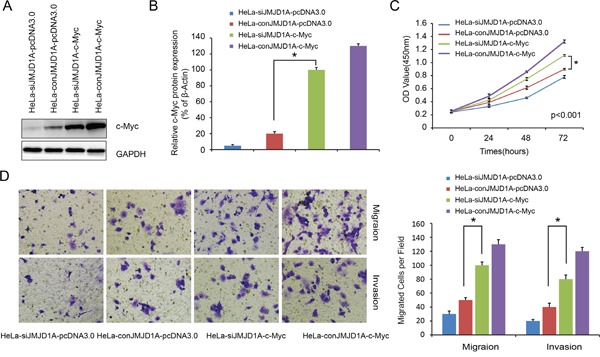
Overexpression of c-Myc rescues the loss of JMJD1A-mediated repression activity in cervical cancer **A.** Western blot analysis was performed to detect the c-Myc expression. We found that c-Myc expression was upregulated in HeLa-siJMJD1A-c-Myc compared to HeLa-conJMJD1A-pcDNA3.0 cell. **B.** Effect of c-Myc overexpression on proliferation was evaluated by Cell Counting Kit-8 assays. The cell proliferation ability was increased in HeLa-siJMJD1A-c-Myc compared to HeLa-conJMJD1A-pcDNA3.0 cell. **C.** Transwell and Boyden chamber assay was performed to examine cell migration and invasion ability. The cell migration and invasion ability was increased in HeLa-siJMJD1A-c-Myc compared to HeLa-conJMJD1A-pcDNA3.0 cell. *P < 0.05.

When we overexpressed c-Myc in HeLa-siJMJD1A and HeLa-conJMJD1A cells, expression of c-Myc was increased in HeLa-siJMJD1A-c-Myc cells as compared to HeLa-conJMJD1A-pcDNA3.0 cells (*P*<0.05, Figure 4A, 4B). HeLa-siJMJD1A-c-Myc cells exhibited increased proliferation, migration, and invasive ability compared to HeLa-conJMJD1A-pcDNA3.0 cells (*P*<0.05, Figure 4C-4E). Thus JMJD1A-induced cervical cancer cell proliferation, migration, and invasion are dependent on c-Myc.

### JMJD1A and c-Myc protein levels correlate with pathologic features of cervical cancer

**Figure 5:**
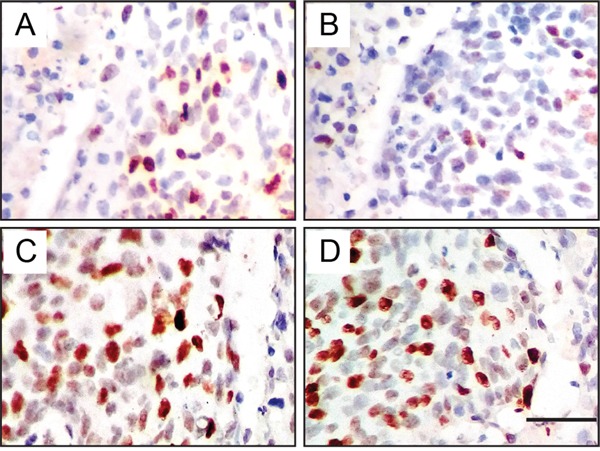
Immunohistochemical expression levels and localization of JMJD1A and c-Myc in cervical cancer tissues Both JMJD1A (A, low; B, High) and c-Myc (C, low; D, High) are localized in the nucleus. Representative pictures show positive correlation of concomitant expression of JMJD1A and c-Myc. **A, C.** Low JMJD1A and c-Myc expression from one patient with FIGO stage I. **B, D.** High JMJD1A and c-Myc expression from another patient with FIGO stage II.

To further confirm this hypothesis that JMJD1A regulates c-Myc transcription, we investigated the expression of JMJD1A and c-Myc in 80 cervical cancer specimens by immunohistochemical analysis. Both JMJD1A (Figure 5A, low; Figure 5B, High) and c-Myc (Figure 5C, low; Figure 5D, High) were localized in the nucleus. Association of JMJD1A and c-Myc expression with clinicopathological factors is summarized in Table 1. Increased JMJD1A expression correlated with lymph node metastasis (*P*=0.031) and FIGO stage (*P*=0.007), while increased c-Myc expression correlated with tumor differentiation (*P*=0.007) and FIGO stage (*P*<0.001). Furthermore, we found a positive correlation between JMJD1A and c-Myc expression (*P*<0.001, Table 2), with Spearman correlation coefficients of 0.397.

**Table 1:** Correlation between the clinicopathologic characteristics and JMJD1A and c-Myc expression in cervical cancer (n = 80)

Variable	Cases	JMJD1A expression		*P*-value	c-Myc expression		*P*-value
		Low(n=34)	High(n=46)		Low(n=38)	High(n=42)	
Age(years)							
<45	32	13	19	0.782	11	21	0.055
≥45	48	21	27		27	21	
Tumor size(cm)							
<4	42	16	26	0.402	20	22	0.982
≥4	38	18	20		18	20	
Histological type							
SCC	62	27	35	0.725	33	29	0.057
NSCC	18	7	11		5	13	
Parametrial infiltration							
No	36	12	24	0.134	14	22	0.163
Yes	44	22	22		24	20	
Differentiation							
Grade 1+2	51	21	30	0.751	30	21	0.007*
Grade 3	29	13	16		8	21	
Lymph node metastasis							
No	67	32	35	0.031*	34	33	0.187
Yes	13	2	11		4	9	
FIGO stage							
<II	31	19	12	0.007*	23	8	<0.001*
≥II	49	15	34		15	34	

SCC, squamous cell carcinoma; NSC, non-squamous cell cancer; FIGO, International Federation of Gynecology and Obstetrics

* *P*<0.05 indicates a significant association among the variables(2-tailed).

**Table 2:** Association between JMJD1A and c-Myc expression in cervical cancer

Tissue sample	c-Myc expression		r	*P*-value
	Low(n=38)	High(n=42)		
JMJD1A Low(n=34)	24	10	0.397	<0.001*
JMJD1A High(n=46)	14	32		

* *P*<0.05 indicates that correlation is significant at the 0.05 level(2-tailed).

### JMJD1A and c-Myc protein levels correlate with survival of cervical cancer patients

**Figure 6:**
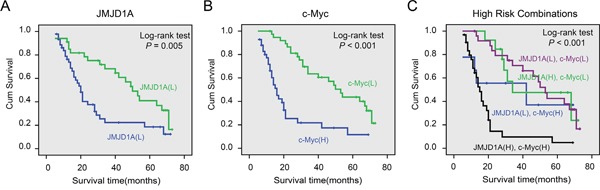
Cumulative Kaplan-Meier overall survival curves of 80 cervical cancer patients segmented by JMJD1A (A), c-Myc (B), and high-risk combination group (JMJD1A and c-Myc combinations) (C) **A.** Compared to JMJD1A low expression group, patients with high levels of JMJD1A had poor survival. **B.** Patients with increased expression of c-Myc had a worse prognosis. **C.** We compared the prognosis with different groups including JMJD1A/c-Myc high/high, low/low, high/low and low/high samples. The results showed that the group with worst prognosis is the high/high group. *P*-values were calculated by the log-rank test. *P < 0.05.

Kaplan-Meier analysis and log-rank test were used to investigate the prognostic value of JMJD1A and c-Myc protein levels and classic clinicopathological characteristics of patients' survival. Using univariate analysis, both JMJD1A and c-Myc protein levels were closely associated with overall survival (OS) of cervical cancer patients (*P*=0.006 and *P*<0.001, Table 3). The log-rank test results showed that increased levels of JMJD1A and c-Myc correlated strongly with poor survival (*P*=0.005 and *P* < 0.001, Figure 6A, 6B).

**Table 3:** Summary of univariate and multivariate Cox regression analysis of overall survival duration in all cervical cancer patients (n = 80)

Clinicopathological parameters	Univariate analysis			Multivariate analysis		
	HR	95% CI	*P*-value	HR	95% CI	*P*-value
JMJD1A(High/Low)	2.229	1.258-3.948	0.006*	1.899	1.025-3.519	0.042*
c-Myc(High/Low)	3.489	1.960-6.211	<0.001*	2.502	1.323-4.731	0.005*
Age(years)(≥45/<45)	0.587	0.338-1.019	0.058			
Tumor size(cm)(≥4/<4)	1.292	0.749-2.228	0.357			
Histological type(NSCC/SCC)	2.402	1.256-4.593	0.008*			
Parametrial infiltration(Yes/No)	0.839	0.484-1.454	0.532			
Differentiation(Grade 3/Grade 1+2)	2.650	1.451-4.839	0.002*	2.317	1.217-4.414	0.011*
Lymph node metastasis(Yes/No)	2.426	1.169-5.035	0.017*			
FIGO stage(≥II/<II)	2.314	1.289-4.153	0.005*			

HR hazard ratio, 95% CI 95% confidence interval

* indicates *P* <0.05.

Univariate analysis indicated that histological type, tumor differentiation, lymph node metastasis, and FIGO stage correlated with patient survival (*P*=0.008, *P*=0.002, *P*=0.017, and *P*= 0.005, respectively). Multivariate analysis showed that JMJD1A and c-Myc expression, and tumor differentiation were independent prognostic factors for cervical cancer patients (Table 3). Histological type, tumor differentiation, lymph node metastasis, and FIGO stage were not associated with survival (Table 3). In addition, the log-rank test showed that strong co-expression of JMJD1A and c-Myc correlated with shorter OS (*P*<0.001, Figure 6C). The 5-year survival rate was only 5% (95% CI, -0.048–0.148) in the high co-expression group and 42% (95% CI, 0.263–0.577) in other groups.

## DISCUSSION

Our study reveals the important role played by JMJD1A in cervical cancer. JMJD1A is upregulated in cervical cancer compared with para-carcinoma. JMJD1A knockdown inhibits cervical cancer cell proliferation, migration, and invasion. Furthermore, JMJD1A induces transcription of c-Myc, which is crucial for cervical cancer growth and progression. Survival analysis reveals that both JMJD1A and c-Myc protein levels are independent prognostic factors for cervical cancer patients. High co-expression of these two proteins correlates with poor prognosis. Our results indicate JMJD1A is a promising therapeutic target in cervical cancer.

JMJD1A is an H3K9me1/2 histone demethylase [10]. Because H3K9 methylation in promoter regions is inhibitory to transcription [22, 23], gene expression tends to be enhanced by JMJD1A activity [24-26]. Moreover, JMJD1A appears to be an important regulator affecting the development and progression of various malignancies. Yamada *et al* [18] reported that JMJD1A is a useful prognostic marker and may enhance malignant transformation in hepatocellular carcinoma. Uemura *et al* [20] showed that JMJD1A is a useful biomarker for hypoxic tumor cells and may be a promising therapeutic target in colorectal cancer. Yang *et al* [19] have shown that JMJD1A suppression inhibits gastric cancer cell proliferation, and suppresses MAPK pathway via transcriptional downregulation of long noncoding RNA MALAT1. JMJD1A-MALAT1-MAPK signaling might participate in the JMJD1A-induced proliferation of gastric cancer cells. Zhan *et al* [27] reported that JMJD1A promotes tumorigenesis and forms a feedback loop with EZH2/let-7c in non-small cell lung cancer.

We observed that JMJD1A levels are increased in cervical cancer tissue as compared to matched non-tumor tissue. In addition, JMJD1A suppression inhibited cervical cancer cell proliferation, migration, and invasion. c-Myc is reportedly upregulated in cervical cancer, suggesting the possibility that c-Myc overexpression drives cervical cancer progression [28, 29]. Here, we showed that JMJD1A binds to the c-Myc promoter and induces c-Myc transcription, which is consistent with earlier findings [21]. In addition, c-Myc overexpression rescues the loss of JMJD1A-mediated repression activity in cervical cancer. More importantly, our observation that c-Myc levels correlated with those of JMJD1A in cervical cancer tissue samples, suggest JMJD1A contributes to c-Myc overexpression in at least some human cervical cancers. Furthermore, elevated JMJD1A and c-Myc levels correlate with poor patient prognosis and survival.

In sum, our findings reveal the oncogenic effects of JMJD1A in cervical cancer and elucidate a possible mechanism by which JMJD1A and c-Myc act to enhance cervical cancer growth and progression. These findings suggest JMJD1A is a potential therapeutic target for the treatment cervical cancer.

## MATERIALS AND METHODS

### Patients and specimens

The study was conducted according to the Declaration of Helsinki and approved by the Ethics Committee of the Second Affiliated Hospital of University of South China. Written informed consent was obtained from all patients. Human fresh cervical cancer tissue samples and adjacent noncancerous control tissues were obtained by surgical resection from ten patients at the Department of Obstetrics and Gynecology, the Second Affiliated Hospital of University of South China. All samples were derived from patients who had not received adjuvant treatment including radiotherapy or chemotherapy prior to surgery. All samples were snap-frozen and stored in liquid nitrogen after collection.

Additionally, a total of 80 paraffin embedded cervical cancer tissue samples were collected from the Department of Obstetrics and Gynecology, the Second Affiliated Hospital of University of South China. The construction of tissue microarray (TMA) was performed by ShGnghGi Outdo Biotech Company (China). The median age of patients was 50 years (ranging from 35 to 75 years old). The overall survival time ranged from 5 to 73 months, with a median of 21 months. Detailed information can be found in Table 1.

### Immunohistochemistry

After deparaffinization and rehydration, TMA sections were subjected 5 minutes to high pressure for antigen retrieval. Endogenous peroxidase activity was blocked using 100 μL of peroxidase block solution for 10 min. The slides were subsequently incubated overnight at 4°C with primary antibodies as follows: JMJD1A (dilution 1:100; Sigma-Aldrich, USA), c-Myc (dilution 1:100, Abcam, Cambridge, MA). Sections were then incubated with biotinylated secondary antibodies (Zymed, San Francisco, CA) for 30 min at room temperature, followed by incubation with streptavidin horseradish peroxidase complex. Finally, sections were incubated with DAB for 2 min. Positive controls were used in each experiment following supplier's instructions. Negative controls using appropriate IgG to replace primary antibody were also run in each experiment (Supplementary Figure 1).

The staining was scored according to the staining intensity as previously described [30]. The percentage of positive cells was scored as follows: 0% (0), 1%–10% (1), 11%–50% (2) and 51%–100% (3). Staining intensity was scored as follows: no staining (0), weak staining (1), moderate staining (2), and strong staining (3). Comprehensive score = staining percentage × intensity. JMJD1A or c-Myc expression was classified as follows: <6 low expression, ≥6 high expression. Immunostaining was independently scored by two pathologists blinded to the clinicopathological characteristics.

### Cell culture

Cervical cancer-derived cell lines SiHa, MDCK, HeLa, C-33A and ME-180, and the non-tumorigenic human epithelial cell line HaCaT were obtained from Cell Bank of Chinese Academy of Science. Cells were cultured in Dulbecco's modified Eagle's medium (DMEM) supplemented with 10% fetal bovine serum (Gibco, Carslbad, CA, USA), at 37°C with an atmosphere of 5% CO_2_ and 90% relative humidity.

### RNA isolation and RT-qPCR analysis

Total RNA was isolated by using miniBEST universal RNA extraction kit (Takara Bio, Inc.) according to the manufacturer's instructions. Reverse transcription and RT-qPCR kits (Takara Bio, Inc.) were used to evaluate mRNA expression of JMJD1A and c-Myc. β-Actin expression was used to normalize for variance. The PCR primer pairs were as follows:

JMJD1A: 5’-CAGGAGCTCCACATCAGGTT-3’ (F), 5’-TGCATCTTTCACTGCATGGT-3’(R). c-Myc: 5’-GGCTCCTGGCAAAAGGTCA-3’ (F), 5’-AGTTGTG CTGATGTGTGGAGA-3’ (R). β-Actin:5’-CGGGAAAT CGTCCGTGACATTAAG-3’(F), 5’-TGATCTCCTTCT GCATCCTGTCGG-3’ (R).

### Western blotting analysis

Cells were lysed in RIPA lysis buffer and the protein concentration was determined by a standard Bradford assay (Beyotime Institute of Biotechnology, Haimen, China). Whole cell extracts were separated by 10% sodium dodecyl sulfate-polyacrylamide gel electrophoresis. Proteins were transferred to polyvinylidene difluoride (PVDF) membranes by Pharmacia Phast gel electrophoresis system (Roche Diagnostics, Indianapolis, IN, USA). The PVDF membrane was blocked with 3% bovine serum albumin for 1 h. The immunoblots were incubated overnight at 4°C with the following primary antibodies: anti-JMJD1A (Sigma-Aldrich, USA), anti-c-Myc (Abcam, Cambridge, MA), and β-actin (Santa Cruz Biotechnology, Inc., Santa Cruz, CA, USA). The membranes were washed, incubated with appropriate secondary antibodies (room temperature, 1 h), and detected using the enhanced chemiluminescence detection system. The data were normalized against β-actin, used as a loading control.

### Lentiviral vector packaging and infection of HeLa cells

Lentiviral vector encoding shRNA JMJD1A was packaged in 293T cells by the calcium phosphate transfection. The supernatants containing lentiviral particles were collected 48 h after transfection (GeneChem, Shanghai, China). HeLa cells were transduced with the supernatant of lentiviral particles in the presence of polybrene (8μg/ml) for 24 h, before replacement with fresh growth media. Polyclonal cells with puromycin resistance were selected for further experiments.

### Plasmid construction and transfection of HeLa cells

Recombinant plasmid pcDNA3.0/c-Myc was constructed by the insertion of human c-Myc cDNA into pcDNA3.0 vector (GeneChem, Shanghai, China). HeLa-conJMJD1A or HeLa-siJMJD1A cells were transfected with pcDNA3.0/c-Myc or pcDNA3.0 vector control. All transfections were carried out using lipofectamine RNAi-MAX (Invitrogen, USA). Transfected cells were analyzed by Western blotting.

### Luciferase reporter assay

Cervical cancer cells were transfected with the indicated c-Myc promoter reporter containing JMJD1A-binding sites (constructed by GeneChem, Shanghai, China). The c-Myc promoter activity was normalized via co-transfection of a β-actin/Renilla luciferase reporter containing a full-length Renilla luciferase gene [31]. The luciferase activity in the cells was quantified using a dual luciferase assay system (Promega) 24 hours after transfection.

### Cell proliferation assay

The effect of JMJD1A knockdown on cell proliferation was determined by measuring absorbance at 450 nm. Briefly, cervical cancer cells in 96-well plates (1×10^3^ cells/well) in triplicate were added (10 μl) to cell counting kit-8 (CCK-8, Dojindo Molecular Technologies, Kumamoto, Japan) and incubated for 24, 48, and 72 h. The absorbance at 450 nm was measured 1 h later.

### Migration and invasion assays

The cell invasive and migratory potential was evaluated using Boyden chamber and Transwell assays, respectively [32]. Briefly, the Boyden chamber assay was conducted using specialized MilliCell chambers, which included a 24-well tissue culture plate with 12 cell culture inserts (Millipore, Bedford, MA, USA). The inserts contained an 8 μm pore size polycarbonate membrane with a pre-coated thin layer of Matrigel (BD Biosciences). Ten percent fetal bovine serum-containing medium was placed in the lower chambers to act as a chemo-attractant. Then, 1×10^5^ cells in a 100 μl volume of serum-free medium were placed in the upper chambers and incubated at 37°C for 36 h. Invasive cells on the bottom surface of the membrane, which had invaded the Matrigel and had migrated through the polycarbonate membrane, were stained by the staining solution, and counted under a microscope in ten randomly selected fields at a magnification of ×200. Transwell assay was the same as the Boyden chamber with the exception that no Matrigel was used and the permeating time for cells was 24 h.

### Chromatin immunoprecipitation (Chip) assay

Chip assays were performed using Imprint Chromatin Immunoprecipitation Kit as described [33], according to the manufacturer's instructions (Millipore, USA). Briefly, cells (5×10^6^) were cross-linked with 1% formaldehyde for 10 min, quenched by addition of 0.125 M glycine, and collected by centrifugation at 800 g for 5 min at 4°C. Cross-linked cells were resuspended in sodium dodecyl sulfonate lysis buffer containing a protease inhibitor cocktail and the soluble chromatin was sheared to fragment DNA. The fragmented chromatin samples were aliquoted as genomic input DNA or immunoprecipitated with JMJD1A antibodies (Sigma-Aldrich, USA) or IgG (Santa Cruz Biotechnology, Inc., Santa Cruz, CA, USA), and incubated at 4°C with rotation overnight. Immunocomplexes were collected by magnetic separator washed, and eluted with Chip elution buffer. DNA was purified on spin columns. The Chip products and genomic input DNA were analyzed by real-time PCR with SYBR Green PCR Master Mix (Applied Biosystems, Foster City, CA). The primer pairs for c-Myc were: 5’-CCAGCGAATTATTCAGAA-3’, 5’-AATTACCATTGACTTCCTC-3’.

### Statistical analysis

All data are presented as the mean ± standard deviation. Means of two groups were compared by Student's t-test; means of three or more groups were compared with one-way analysis of variance using SPSS for Windows version 13.0 (SPSS, Inc., Chicago, IL, USA). The χ^2^ test was used to analyze the correlation between the clinicopathological characteristics and JMJD1A and c-Myc expression. Overall survival (OS) was defined as the interval from date of diagnosis until death from any cause. Data were analyzed for living patients and patients lost between follow-ups. The OS was estimated using the Kaplan-Meier method and compared using the log-rank test. Significant variables were further analyzed by multivariate analysis to test for independent prognosis. Bivariate correlations between variable factors were calculated by Spearman rank correlation coefficients. *P*<0.05 was considered to indicate a statistically significant difference.

## SUPPLEMENTARY MATERIALS FIGURE


