# Supplementary material for: Jumonji domain-containing protein 1A promotes cell growth and progression via transactivation of c-Myc expression and predicts a poor prognosis in cervical cancer
Source: Oncotarget. 2016 Nov 8;7(51):85151–62. doi: 10.18632/oncotarget.13208 (PMC5356725; doi:10.18632/oncotarget.13208)
Supplement: Supplementary file 1 [file oncotarget-07-85151-s001.pdf]

Jumonji domain-containing protein 1A promotes cell growth and progression via transactivation of c-Myc expression and predicts a poor prognosis in cervical cancer

SUPPLEMENTARY FIGURE

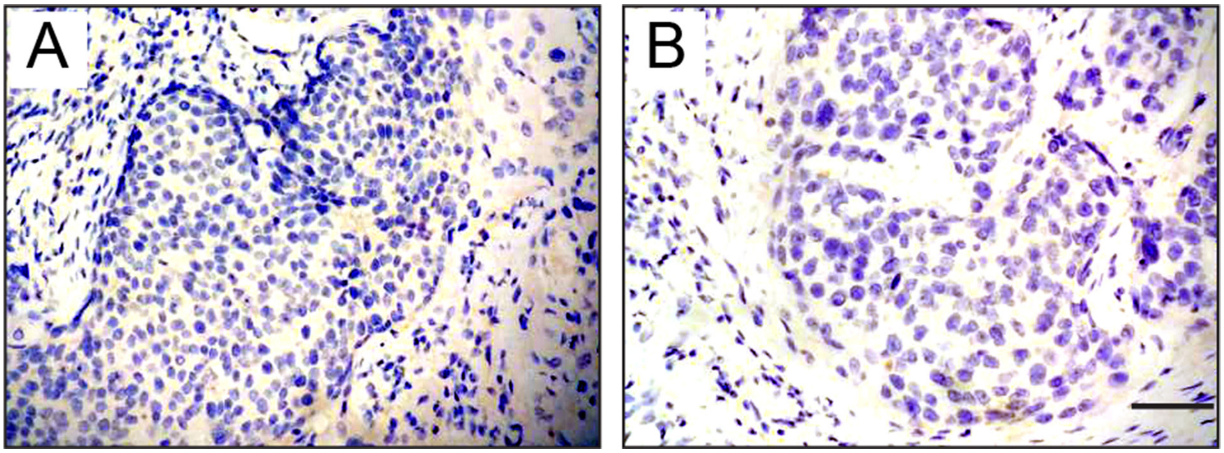

Supplementary Figure S1: Immunohistochemical staining of negative control for JMJD1A A. and c-Myc B. in cervical cancer tissues. Scale bar, 50 μm.
